# MDMA-assisted PTSD and Alcohol Therapy Trial (MPATHY): study protocol for a double-blind, randomised, controlled outpatient trial of MDMA-assisted integrated exposure-based therapy for comorbid post-traumatic stress disorder and alcohol use disorder

**DOI:** 10.1136/bmjopen-2025-114896

**Published:** 2026-07-06

**Authors:** Kirsten C Morley, S Arunogiri, K Mills, J Watt, M Teesson, A Baillie, Y Y Lee, A Morse, S E Back, D I Lubman, P S Haber

**Affiliations:** 1 Specialty of Addiction Medicine, Sydney Medical School, Faculty of Medicine and Health, University of Sydney, Sydney, New South Wales, Australia; 2 Edith Collins Centre for Translational Research, Royal Prince Alfred Hospital, Sydney Local Health District, Sydney, New South Wales, Australia; 3 Eastern Health, Turning Point, Melbourne, Victoria, Australia; 4 Monash Addiction Research Centre, Eastern Health Clinical School, Faculty of Medicine, Nursing and Health Sciences, Monash University, Melbourne, Victoria, Australia; 5 The Matilda Centre for Research in Mental Health and Substance Use, Faculty of Medicine and Health, University of Sydney, Sydney, New South Wales, Australia; 6 School of Health Sciences, Faculty of Medicine and Health, University of Sydney, Sydney, New South Wales, Australia; 7 Health Economics Group, School of Public Health and Preventative Medicine, Monash University, Melbourne, Victoria, Australia; 8 School of Public Health, The University of Queensland, Brisbane, Queensland, Australia; 9 Queensland Centre for Mental Health Research, Brisbane, Queensland, Australia; 10 National Centre for Mental Health Research, Australian National University, Canberra, Australian Capital Territory, Australia; 11 Department of Psychiatry and Behavioral Sciences, Medical University of South Carolina, Charleston, South Carolina, USA

**Keywords:** Substance misuse, Stress Disorders, Traumatic, Acute, Anxiety disorders

## Abstract

**Introduction:**

The treatment of comorbid post-traumatic stress disorder (PTSD) and alcohol use disorder (AUD) is significantly more challenging than the treatment of either disorder alone. While gold standard evidence-based treatments exist for this comorbidity, clinically significant improvements are only observed in approximately half of clinical trial participants. The use of adjunctive pharmacotherapies, such as 3,4-methylenedioxymethamphetamine (MDMA), may serve to optimise gold standard interventions. The primary aim of the MDMA-assisted PTSD and Alcohol Therapy Trial study is to examine the therapeutic and cost-effectiveness of combining MDMA with evidence-based integrated care for comorbid PTSD+AUD. Specifically, we will examine MDMA-assisted integrated exposure therapy versus active control-assisted integrated exposure therapy in improving treatment outcomes for PTSD+AUD.

**Methods and analysis:**

This world-first double-blind trial will aim to randomise 100 participants with PTSD+AUD to a regimen of Concurrent Treatment of PTSD and Substance Use Disorders Using Prolonged Exposure (COPE) (12 sessions)+MDMA (80–160 mg: 2 dosing and 2 integration sessions) or COPE (12 sessions)+active control (niacin 250 mg: 2 dosing sessions and 2 integration sessions). All participants will receive medical management. The primary PTSD outcome will be the clinician-administered PTSD Scale for DSM-5. The primary drinking outcome will be the number of heavy drinking days (HDDs) per week, validated by phosphatidylethanol. Secondary PTSD and alcohol-related outcomes will include PTSD checklist for DSM-5 scores, absence of any HDDs and standard drinks per drinking day. We will also examine change in other clinical conditions and symptoms including depression, sleep disturbances and post-traumatic cognitions; treatment satisfaction and engagement; adverse events; and cost-effectiveness.

**Ethics and dissemination:**

This study will be conducted in accordance with the ethical principles outlined in the Declaration of Helsinki and the International Conference on Harmonisation-Good Clinical Practice guidelines. Ethical approval has been granted by the Sydney Local Health District Ethics Review Committee (X22-0121 & 2022/ETH00773). The results of this study will provide world-first data regarding safety, efficacy and cost-effectiveness of MDMA to optimise integrated exposure-based therapy for comorbid AUD and PTSD and will be disseminated to ensure wide accessibility and to support further research and clinical application.

**Trial registration number:**

NCT05709353.

**STRENGTH AND LIMITATIONS OF THIS STUDY**

- A strength of the MDMA-assisted PTSD and Alcohol Therapy Trial (MPATHY) trial is that it targets a common and challenging comorbidity (post-traumatic stress disorder (PTSD) with comorbid alcohol use disorder) that is not addressed in the majority of other 3,4-methylenedioxymethamphetamine (MDMA)-assisted therapy trials for PTSD.
- The MPATHY trial will minimise bias due to the use of the gold standard objective biomarker phosphatidylethanol to validate alcohol consumption data.
- The MPATHY trial will minimise bias due to the randomised and double-blind design in addition to the inclusion of an active control and prospective stratification for expectancy.
- The MPATHY trial is that it leverages an evidence-based behavioural intervention with strong empirical support for all participants.
- A potential limitation of this trial is the comprehensive nature of the evidence-based intervention combined with MDMA which may be resource intensive.

## Introduction

 Alcohol use disorder (AUD) is among the most prevalent and burdensome mental health conditions worldwide.^1^ For approximately 30%–40% of treatment-seeking individuals with AUD, the course of their illness, and attempts at recovery from AUD, are complicated by comorbid post-traumatic stress disorder (PTSD),^2^ a chronic and debilitating disorder that may develop following exposure to extreme trauma such as combat, sexual and physical assault, disasters and life-threatening accidents.^3^ This rate is alarmingly high when compared with the general population prevalence of 4%.^4^ The elevated prevalence of PTSD among people with AUD, and vice versa, is of considerable clinical concern due to the profound social, health and economic consequences of this comorbidity. Individuals with this comorbidity can often experience a more severe and complex form of PTSD characterised by extensive trauma histories, substantial clinical impairment,^5^ poor prognosis, psychosocial problems, substantially elevated suicide risk^6^ and high treatment attrition^7^ when compared with people with either disorder alone. Clinicians also view the treatment of this common comorbidity to be significantly more challenging than the treatment of either disorder alone.^8^

Underlying the relationship between these disorders are neural substrates common to both AUD and PTSD, including dysregulation of the body’s biological stress response system involving the hypothalamic-pituitary-adrenal axis and the noradrenergic/sympathetic system.^9^ In many cases, the AUD develops, and is maintained by, repeated attempts to ‘self-medicate’ PTSD symptoms^10^; in particular symptoms of avoidance and/or hyperarousal. Although alcohol use may provide short-term relief from symptoms by inhibiting central noradrenergic activity and its associated arousal states,^11^ growing tolerance to its effects leads to increasing use to obtain sufficient PTSD symptom reduction. In the absence of alcohol, PTSD symptoms may worsen, making it difficult, and in many cases impossible, for those experiencing these conditions to maintain abstinence or reduced use. Thus, once established, a cyclical relationship is formed whereby both disorders serve to maintain and exacerbate the other, leading to a chronic course of illness and significant treatment complications. Siloed approaches to the treatment of AUD and PTSD, whereby each disorder is treated sequentially, are largely ineffective.^12^

Systematic reviews and meta-analyses point to the safety and efficacy of integrated treatments that combine exposure-based cognitive behavioural therapies (CBTs) for AUD and PTSD.^13^,^15^ The approach with the most evidence to date is Concurrent Treatment of PTSD and Substance Use Disorders Using Prolonged Exposure (COPE).^16^ COPE combines prolonged exposure therapy to treat PTSD (imaginal exposure to trauma-related memories and in vivo exposure to avoided situations) with treatment components for alcohol use such as motivational enhancement therapy (eg, improving motivation) and CBT (eg, exploring how thoughts affect our behaviour, challenging maladaptive cognitions, managing cravings). Several randomised controlled trials (RCTs) have demonstrated that COPE is safe and effective in producing substantial reductions in both PTSD and AUD symptom severity, even among individuals with complex trauma histories and severe substance use disorders.^17^,^20^ COPE was designated as a first-line treatment for PTSD and substance use disorders by the American Psychological Association.^21^ Yet even when provided gold standard care such as COPE, only about half of participants in clinical trials demonstrate clinically significant improvements.^15 22^ Impediments to achieving optimal outcomes include difficulties engaging with critical components of exposure therapy, such as the capacity to recall, emotionally and cognitively engage with and process traumatic memories. The use of adjunctive pharmacotherapies may serve to unlock these barriers and optimise gold standard interventions^23^ through activation and deactivation of common neural pathways and has been identified as a priority by the International Society for Traumatic Stress Studies.^24^

In particular, 3,4-methylenedioxymethamphetamine (MDMA) is a promising candidate pharmacotherapy for augmenting COPE. MDMA raises levels of monoamine neurotransmitters in the brain (serotonin, norepinephrine, dopamine) and hormones (oxytocin, cortisol) leading to enhanced mood and increased feelings of trust and closeness to others,^25^,^28^ which may potentiate a stronger therapeutic alliance between client and clinician which is essential to all psychotherapies and may be particularly helpful in the context of PTSD and substance use.^29 30^ By reducing activation in the amygdala and insula and increasing connectivity between the amygdala and hippocampus, MDMA may enhance a person’s ability to retrieve, approach and emotionally engage with, process and reconsolidate traumatic memories and facilitate fear extinction.^29^ These mechanisms, while not specifically examined in the current trial, are key to the effectiveness of exposure therapy and are particularly challenging for individuals with comorbid AUD. Clinical trials examining the efficacy of MDMA-assisted therapy for PTSD suggest that MDMA-assisted therapy could be a promising candidate for augmenting treatment outcomes for individuals with PTSD.^31^ MDMA is administered in a controlled clinical setting as an adjunct to psychotherapy, combining preparatory sessions, dosing sessions and integration sessions. Integration sessions most often occur the day following dosing (sometimes referred to as the ‘therapeutic window’), with the aim of processing insights, emotions and memories that arose during the dosing session. With regards to AUD, there has been just one open-label pilot study investigating MDMA-assisted therapy in 14 patients with AUD which reported that MDMA was well tolerated and reduced self-reported alcohol consumption^32^ in addition to reduced cravings and improved sleep.^33^ However, the small sample, lack of a control group and objective outcomes indicate that these findings are exploratory and require further replication. Taken together, there is solid rationale and emerging clinical data suggesting that MDMA-assisted therapy could be of therapeutic benefit for individuals suffering from these disorders concurrently.

Accordingly, we aim to conduct a world-first double-blind RCT of an evidence-based integrated exposure therapy+MDMA for the treatment of comorbid PTSD+AUD: The MDMA-assisted PTSD and Alcohol Therapy Trial (MPATHY). We aim to address well-documented concerns pertaining to the methodological designs associated with the psychedelic-assisted therapy literature^34^ by including an active control, prospective stratification for expectancy and utilisation of evidence-based therapeutic modalities for comorbid PTSD+AUD as the ‘base’ therapy. Indeed, this will be the first randomised double-blind controlled trial of MDMA-assisted therapy that uses a therapy that has well-documented effectiveness for this specific comorbidity (ie, COPE).

We hypothesise that in participants with comorbid PTSD+AUD, MDMA-assisted integrated exposure therapy relative to active control-assisted integrated exposure therapy will: (1) significantly reduce the severity of PTSD symptoms; (2) significantly reduce alcohol consumption; (3) significantly improve depression, insomnia and quality of life; (4) yield good treatment satisfaction and engagement; (5) have minimal adverse events (AEs) and (6) be cost-effective. This article presents the protocol for the RCT and is written to comply with the recommended Standard Protocol Items: Recommendations for Interventional Trials (SPIRIT) guidelines for RCT protocols.^35^

## Methods

### Study objectives

This is a double-blind RCT to examine the effectiveness of MDMA-assisted COPE therapy versus active control-assisted COPE therapy in improving treatment outcomes for individuals with PTSD+AUD.

### Study design

**Figure 1:**
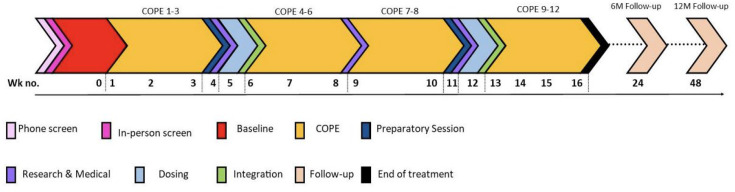
Flow chart of trial procedure for a double-blind RCT of an evidence-based integrated exposure therapy+MDMA for the treatment of comorbid PTSD+AUD. The MDMA-assisted PTSD and Alcohol Therapy Trial (MPATHY). AUD, alcohol use disorder; COPE, Concurrent Treatment of PTSD and Substance Use Disorders Using Prolonged Exposure; M, month; MDMA, 3,4-methylenedioxymethamphetamine; PTSD, post-traumatic stress disorder; RCT, randomised controlled trial.

This study is a parallel-group RCT conducted at two clinical sites. Following consent, pre-treatment screening and assessment, and randomisation, participants will receive 12 sessions of integrated exposure therapy (COPE) and two dosing sessions (MDMA or control) at visits 5 and 12. Each dosing session is preceded by a preparatory session the week prior and followed by an integration session the day after. Thus, participants in both arms may receive up to 18 sessions of therapy in total (see figure 1). Follow-up visits will occur at the final COPE session (end of treatment), and 6 and 12 months after baseline. The design follows Good Clinical Practice, Consolidated Standards of Reporting Trials and SPIRIT guidelines.

### Participant recruitment

The trial is being conducted in two outpatient hospital and community settings in New South Wales (NSW) and Victoria (VIC), Australia. The trial is now in the recruitment phase (expected completion: 2027). We aim to recruit participants through clinical referral (from treating clinicians of participating hospitals and community clinics) and via flyers/community advertisements in general practices, newspapers, websites and social media (eg, Facebook). Based on our previous experience, recruitment from mixed sources does not significantly impact treatment retention or outcome in alcohol trials.^36^ Participant time and travel expenses are reimbursed at the follow-up research assessments.

### Randomisation and allocation concealment

The study is a double-blind, RCT with participants randomised to receive one of two possible medication arms, in addition to COPE therapy. The study is conducted under double-blind conditions so that participants and study staff are unaware of medication assignment. A computer-generated random allocation to MDMA or active control (niacin) will be provided by an independent service and conducted in a secure web-based database (RedCap software) and stratified for balancing by site (NSW vs VIC), sex at birth (male vs female), prior MDMA use (Y/N), prior selective serotonin reuptake inhibitor use (Y/N), PTSD severity (PTSD checklist for DSM-5 (PCL-5): >47), trauma (multiple vs single) and treatment expectancy (positive vs negative, Stanford Expectations of Treatment). This information is provided to the Clinical Trial Pharmacists at each site for intervention allocation and medication dispensing. In the event of a medical emergency that requires knowledge of the treatment condition, the investigators can contact the pharmacist to break the randomisation code for that individual.

### Inclusion and exclusion criteria

#### Inclusion criteria

(a) Both current PTSD and AUD according to the DSM-5 criteria, for 6 months or longer as assessed via Clinician-Administered PTSD Scale for DSM-5 (CAPS-5) and the Structured Clinical Interview for DSM-5 – Research Version (SCID-5-RV); (b) age >18 years; (c) adequate cognition and English language skills to give valid consent and complete research interviews and assessments; (d) willing to give written informed consent; (e) received prior treatment for PTSD or AUD such as approved psychosocial or pharmacological interventions (given MDMA-assisted therapies are designed to target those that do not respond to traditional therapies); (f) stable housing and (g) able to identify a significant other (such as a family/friend/partner) who could accompany them from the clinic, provide transport and/or be contacted by the study team if required.

#### Exclusion criteria

(a) History of, or currently meeting DSM-5 criteria for a current or lifetime psychotic or bipolar disorders, or major depression with psychotic features, as assessed via the SCID-5; (b) pregnant or lactating (contraception must be used and a sensitive pregnancy test will be performed at baseline and prior to dosing); (c) significant alcohol withdrawal (Clinical Institute Withdrawal Assessment for Alcohol (CIWA-Ar) score ≥10, including history of delirium tremens or alcohol withdrawal seizures); (d) concurrent use of psychotropic medication (antidepressants and alcohol pharmacotherapy use considered for inclusion if assessed by physician and titrated down with 5 half-lives+1 week washout); (e) use of, and unable or unwilling to cease, any medications likely to interact with MDMA in the opinion of the physicians and investigators during the trial (low dose opiates are permitted for pain management but not the night before or after MDMA sessions); (f) current substance use disorder (DSM-5) other than tobacco; (g) abnormal clinical findings including a history of, or current: cardiac disease and/or dysrhythmia, uncontrolled hypertension or severe hypotension, abnormal ECG findings, stroke, liver disease, a history of epilepsy, hyponatraemia or malignant hyperthermia (controlled hypertension and diabetes type II may be permitted) and (h) suicide risk according to clinician judgement and responses to Columbia Suicide Severity Rating Scale-Lifetime (C-SSRS-L) and SCID-5-RV. Details surrounding any previous suicide attempts will be gathered whereby attempts related to their trauma/PTSD and/or associated with the use of psychostimulants will contribute to risk assessment and guide trial safety measures if enrolled; (i) clinically unstable systemic medical (eg, cancer) or psychiatric disorder or condition that might require hospitalisation that precludes trial participation; (j) regular use of ecstasy (at least twice in last 6 months or >10 times within the last 5 years) and (k) enrolled in any other interventional clinical trials in the previous 2 months or over the duration of the study.

### Procedures and schedule of visits

Participants are initially pre-screened either face-to-face by their treating clinician or over the phone by a psychologist or nurse following either self-referral or referral by a medical officer or psychologist. If clinically indicated, participants are offered alcohol withdrawal management services. Eligible participants are then offered further onsite screening for a full eligibility assessment composed of comprehensive medical and psychiatric assessment. The schedule of enrolment, interventions and assessments are depicted in online supplemental table 1.

*Screening visits a and b:* At the onsite screening visits (a and b), participants are asked to provide informed consent by the research nurse. The Participant Information Sheet and Consent Form (PISCF) given to participants clearly outlines the study aims, what is required of the participant and any benefits or risks to participation. Potential participants will be given ample time to review the PISCF and ask questions of their family, friends and/or doctors, prior to providing informed consent. The medical officer performs a physical examination and obtains a structured medical and psychiatric history including management plan for tapering of contraindicated concomitant medications where necessary. Blood and urine samples are taken for clinical laboratory evaluations (see below). The psychologist obtains a structured psychological assessment (see below).

*Baseline visit:* after screening and, if necessary, concomitant medication management is completed, eligible participants undergo a baseline research evaluation.

*Visits 1–3, 6–10, 13–16 (including COPE treatment sessions):* participants receive COPE therapy (see below) delivered by the psychologist the week following their baseline visit and return weekly for sessions. Concomitant medications and AEts (see below) are documented at these visits.

*Visits 8, 16 (medical & research visit):* blood samples are taken for clinical evaluation (liver function tests (LFTs)) and research (phosphatidylethanol (PEth)).

*Visits 4 and 11 (preparation visit):* participant attends a session with the psychologist and co-therapist to prepare for dosing session (see below). Medical review is conducted in view of dosing sessions including examining recent LFTs, concomitant medication and AEs.

*Visits 5a and 12a (dosing visits):* participant attends the dosing session with the psychologist and co-therapist which includes monitoring by the physician and nurse (see below).

*Visits 5b and 12b (integration visits):* participant attends the integration session with the psychologist and co-therapist which includes monitoring and assessment by the nurse (see below).

*Visits 16–18 (follow-up visits):* participants return for the end of treatment review and research follow-up immediately after the final COPE session (visit 16) and then for 6 and 12 follow-up research appointments (visits 17 and 18).

### Medications

Participants will be randomly assigned before dosing to the MDMA or control arm. During dosing sessions, participants randomised to: (1) the MDMA arm will receive MDMA plus placebo (matched to niacin) or (2) the control arm will receive placebo (matched to MDMA) plus niacin. The dosage of MDMA is 80–120 mg MDMA HCl (Dose 1) and 80–160 mg MDMA HCl (Dose 2). PharmAla Biotech, in Canada, will source MDMA (40 mg capsules of MDMA HCl) from Dalton Pharma Services, a good manufacturing practice (GMP)-licenced facility. Placebo contains mannitol (98% w/w), magnesium stearate (2% w/w) and will be sourced by PharmAla Biotech from Psilo Scientific (GMP-licenced). MDMA will be over-encapsulated in gelatine capsules containing mannitol and magnesium stearate. The dosage of active control, niacin (nicotinamide), is 250 mg and will be sourced from Syntro and over-encapsulated with microcrystalline cellulose to look identical to a niacin placebo. Medications are over-encapsulated to maintain the double-blind. A check on the blind is obtained throughout (mid-point and end of treatment) by asking the participant, clinician and the researcher to record the study drug the participant is thought to have received (active drug or active control). The proportion that guessed allocation correctly will be reported in the final results and a sensitivity analysis conducted to determine whether outcomes differ by guessed allocation.

Prior to the dosing sessions, participants will discuss an optional supplemental dose of the study drug with their clinicians. This additional dose will be administered approximately 60–90 min after the initial dose on each dosing day. A singular supplemental dose per dosing session will only be available if: (1) both participant and clinician agree prior to the dosing day, day of dosing and post-initial dose; (2) prior dose was well-tolerated and/or if there are no AEs of concern in the first hour post-dosing, with the AEs of concern being determined by clinician judgement and (3) participant’s vital signs (blood pressure (BP) and heart rate (HR)) remain within standard observational requirements (eg, BP <180/110) or as per clinician judgement (eg, when between BP 160–180/110). Supplemental doses are optional and can be declined by participants or investigators and clinicians at any time. This supplemental dosing schedule is aligned with potential variations in tolerability due to pharmacokinetics and pharmacodynamics and is consistent with the majority of published trials globally.^37^

### Integrated prolonged exposure therapy

COPE represents an integration of existing evidence-based manualised CBT interventions for PTSD and substance use disorders, including imaginal and in vivo exposure, delivered over 12 individual 90 min sessions (total of 19.5 hours).^38^ Although designed to be delivered weekly, flexibility is permitted. More details on COPE session procedures are available in the published *COPE Manual*.^38^ COPE therapists will be Masters-level (or higher) clinical psychologists with extensive training and experience. All study therapists will be involved in regular meetings and receive clinical supervision to discuss participant adherence to treatment and clinical concerns about participants, provide feedback to reduce departure from protocol and assist therapists in identifying issues for subsequent sessions. Therapy sessions will be audio recorded for research and training purposes and randomly selected sessions (25%) will be evaluated with the Yale Adherence and Competence Scale II^39^ to ensure consistency with manual guidelines. Interrater reliability on adherence and competence measures and intraclass correlation coefficients will be calculated. Abstinence from alcohol will be encouraged but not required. Following each session, the participant will complete a brief post-session rating with the Session Rating Scale (SRS).^40^ Recordings of the sessions will be evaluated by the therapists using the Motivational Areas Rating Scale.^41^

### Preparation, dosing and integration sessions

Here we briefly outline the MPATHY preparatory, dosing and integration sessions. More detail is provided in the *MPATHY Therapeutic Dosing Manual* (available on request).

In the preparation component, the COPE therapist is joined by a trained co-therapist. This dyad will then: (1) discuss the details for the day procedure such as length, music, dosing administration procedure, medical monitoring, boundaries of interaction and safety measures; (2) provide some psychoeducation regarding the medications (active control and MDMA); (3) describe the therapeutic approach; (4) revisit techniques from the COPE sessions relevant for the dosing session and (5) explore and clarify the participant’s expectations, intent and hopes of the dosing session including managing expectancy issues, anxieties and alcohol consumption.

Dosing sessions will be conducted in a quiet room with soft ambient lighting and comfortable furnishings. The air-conditioning will be set to an ambient temperature of 20–24°C to ensure a comfortable and safe level. A music soundtrack from WavePath (https://wavepaths.com) will be available. These sessions will be approximately 6 hours in duration. It is a requirement that all participants have a 0.00% breath alcohol concentration at the start of the session or the session will be postponed. Dosing sessions will be audio and video recorded for safety, research and training. Training for these sessions will be provided by clinicians/therapists with experience delivering psychedelic-assisted therapy in clinical trials and clinical supervision. Two people will always be present in the room with the participant on dosing sessions including the COPE-trained clinical psychologist and another appropriately trained co-therapist or staff member (eg, physician, psychologist, experienced research nurse or peer worker). These sessions will thus be unstructured with no therapist-led direction regarding trauma; therapists will keep comments to a minimum and not speak much (to avoid interfering) unless invited by the participant (staying within MDMA-assisted therapy and COPE-aligned therapeutic approaches). All sessions will be audio and video recorded for safety, research and training purposes and will be audited for quality control (eg, fidelity with scripts, language bias re expectancy, etc). Light refreshments will be made available according to a standardised menu plan including sandwich, biscuits and mild/decaffeinated tea or coffee and water (fluid intake will remain <3 L and be spread out appropriately during the session).

The day after each dosing session, participants will attend an ‘Integration Session’ in person with the same dyad. The aim of these sessions is to reflect on the session and discuss the content of the dosing session. More specifically, the Integration Session will entail: exploring and processing cognitions, emotions, sensations and memories that arose during the dosing session; reflecting on key themes; and supporting incorporation into the goals and intention of ongoing therapy aligning with the principles of COPE.

### Study assessments

See online supplemental table 1 for a comprehensive list of study assessments. We will attempt to obtain data for all randomised participants regardless of whether they discontinue treatment and study visits as per intention to treat. Follow-up data may be obtained by telephone.

#### Medical/laboratory tests

These tests screen participants for exclusion criteria, assess potential AEs, provide objective measures of alcohol use and assess covariates and secondary outcome measures. The physical examination at screening includes BP, HR, ECG, height, weight, full blood count, LFTs (bilirubin, Gamma-Glutamyl Transferase, Alkaline Phosphatase, Aspartate Aminotransferase, Alanine Aminotransferase, albumin, protein), coagulation (International Normalised Ratio, Activated Partial Thromboplastin Time), urine toxicology, concomitant medications, withdrawal symptoms (CIWA-Ar). All abnormal findings will be reviewed by the physician, and participation is endorsed or further evaluation performed as clinically indicated. Tests will be repeated if clinically indicated and for baseline measurement if required. PEth is collected at screening/baseline, mid-point (visit 8) and end of treatment (visit 18). Blood samples will also be collected and stored for biochemical and genetic exploratory analyses relating to the current trial and for future use in ancillary studies.

#### Questionnaires and tasks

Demographics, medical history, personal and family history of PTSD and AUD and relevant treatment history will be obtained during screening as in our previous trials.^42^,^47^ The CAPS-5 will be used to obtain diagnosis of PTSD and symptom severity^48^ and the Life Events Checklist for DSM-5 will be used to measure lifetime exposure to potentially traumatic events.^48^ The SCID-5-RV^49^ will be used to obtain AUD diagnosis and gather psychiatric diagnostic information relevant for screening. The C-SSRS-L will be used to screen for suicidal risk in addition to clinician assessment.^50^ The Montreal Cognitive Assessment will be used to screen for cognitive impairment likely to preclude completion of study procedures and treatment (≤24/30 excluded).^51^

The following measures will be implemented at baseline, mid point (visit 8) and end of treatment follow-up: (1) recent (last 30 days) alcohol consumption (frequency/quantity) assessed by the Timeline Follow-Back method (TLFB)^52^; (2) severity of alcohol dependence assessed by the Alcohol Dependence Scale^53^; (3) symptoms of PTSD will be assessed by the PTSD checklist for DSM-5 (PCL-5^48^); (4) severity of symptoms of depression, anxiety and stress assessed by the Depression, Anxiety and Stress Scale (DASS)^54^; (5) recent use of health services assessed by the Health Service Use Questionnaire; (6) sleep disturbance assessed by the Insomnia Severity Index^55^ (ISI); (7) dysfunctional cognitions that may develop following trauma measured by the Post Traumatic Cognitions Inventory (PTCI^56^) and (8) health-related quality of life measured by the Short Form 36 (SF-36^57^). At baseline, major domains of personality will be measured with the NEO Personality Inventory 3^58^; adverse or traumatic childhood experiences will be measured by the adverse childhood experience scale (ACE^59^) and treatment expectancy will be assessed using the Stanford Expectations of Treatment Scale.^60^ The revised Mystical Experience Questionnaire 30^61^ will be administered after dosing sessions to examine mystical, positive mood, ineffability and the Psychedelic Music Questionnaire Short Form will be administered during integration sessions to assess the participants’ experience of music during dosing sessions. At baseline and end of treatment follow-up we will also administer: (1) The Trail Making Test^62^ to measure the speed of processing and executive functioning; (2) Number-letter task (adapted from Rogers and Monsell^63^) to assess working memory; (3) the Emotional Stroop Test will be used to measure attentional biases toward emotional stimuli^64^ and the (4) California Verbal Learning Task to measure learning and retrieval strategies for verbal material.^65^

The TLFB, PCL-5 and C-SSRS will be repeated at each clinic visit (ie, COPE therapy sessions and preparation and integration sessions) to monitor alcohol consumption, symptom improvement and suicide risk. We will also collect daily drinking and mood using Positive and Negative Affect Schedule (PANAS^66^) via Smartphone Ecological Momentary Assessment (SEMA3) for 7 days following the baseline visit and the two dosing sessions.

The SRS will be used in COPE and dosing sessions to measure the quality of the therapeutic relationship and experience of the session.^67^ Therapeutic alliance will be measured by the Helping Alliance Questionnaire^68^ at several time points (visits 4, 8, 11, 16). At end of treatment follow-up, treatment satisfaction will be measured using the Client Satisfaction Questionnaire (CSQ^69^) and the Your Experience of Service (YES^70^) measure.

#### AEs monitoring and management

All AEs reported between consent and final follow-up will be recorded in the electronic case report form. At each visit, the C-SSRS-R will be administered to monitor suicidality, PCL-5 to monitor PTSD symptoms and mood and TLFB to monitor alcohol use. Participants will also be asked at each visit an open question regarding any new physical or mental health symptoms.

During dosing, a medical practitioner and nurse will be on call and participants will be monitored throughout the session according to following: vital signs (BP, HR and body temperature (BT)) taken regularly throughout the dosing session; regular observations aligning with a standardised list used in previous MDMA studies^71 72^; hourly assessment of mood (visual Likert scale 0–10).

During dosing sessions, the following AEs will be addressed accordingly: (1) temperature of >38.5°C: active cooling will commence. This may include fan and/or with ice/cool packs to the groin and axilla. Additional vital sign measurements where appropriate in consultation with the site physician; (2) chest pain, shortness of breath, neurological deficit or confusion: Additional vital sign measurements and intervention where appropriate in consultation with the site physician. Participants would be referred to the hospital emergency department if cardiac or cerebral ischaemia is suspected. Participants experiencing such symptoms would be excluded from subsequent dosing sessions; (3) development tremor or abnormal movements: physician assessment. Medical management of any other AEs will be addressed as per standard procedures of the requisite health setting with additional monitoring and intervention from site physician. Non-medication methods for managing any anxiety will be emphasised. Prior to discharging the participant, BP, HR and BT will be measured. Participants will be discharged when safe and will be escorted home by a nominated significant other (eg, partner/friend/family member). If the participant expresses the desire to leave and cannot gently be encouraged to stay within the designated therapy timeframe they will be medically assessed for safety. If deemed unsafe to leave, they will be requested to stay on routine clinical grounds until they are safe to leave. Overnight hospital stay will be available if clinically indicated. Participants will be advised not to drive or operate machinery for 48 hours after dosing. Participants must refrain from using low dose opiates for pain management for the evening post-dosing. If a participant exhibits any signs of toxicity, they will not receive another dosing session. If the participant reports adverse psychological symptoms, they are not required to receive another dosing session.

In the day following each dosing session (integration) and at the next visit, AEs will also be collected using the Systematic Assessment for Treatment Emergent Effects (SAFTEE) interview,^73^ a widely used measure of AEs and for which a standardised approach exists for trials of medication for AUD. At the integration session symptoms such as mood, alcohol consumption, sleeping and suicidality are monitored. During the week following each dosing session, regular phone calls with participants will discuss any AEs or changes to concurrent medications. Participants will also be asked to complete the PANAS three times a day for the week and record alcohol consumption using the SEMA software. Desire to use illicit ecstasy will be monitored with specific questions pertaining to this issue asked in the following COPE sessions.

#### Data and safety monitoring

AEs are collected at every study visit and the site investigator rates their relationship to medication. Serious AEs are defined as by the Therapeutics Goods Administration.^74^ If serious or unexpected AEs occur during the trial, they are reviewed by the principal investigator and the trial team and reported within the specified time frames to the lead hospital ethics review committee and to the Data and Safety Monitoring Board (DSMB). The DSMB comprises three members independent of the research study and funding body and meets regularly to provide oversight and monitoring to ensure participant safety and trial validity and integrity. The DSMB can request that the trial be terminated by the principal investigator in the event of safety concerns. Participants are assigned a code and their information de-identified in password protected documents.

#### Outcomes

##### Primary

The primary outcome is PTSD symptom severity as assessed by the CAPS-5 from baseline to end of treatment (visit 16). CAPS-5 is a structured diagnostic interview with excellent psychometric properties and diagnostic efficiency and used widely in MDMA-assisted PTSD studies. The CAPS-5 will be administered by independent evaluators blind to treatment condition. We will also examine secondary PTSD outcomes including change in self-reported PTSD symptom severity via the PCL-5 from baseline to visit 16. PCL-5 has excellent psychometric characteristics for a secondary indicator of PTSD symptom severity.

##### Secondary

The secondary outcome will be alcohol consumption expressed as self-reported number of heavy drinking days (HDDs) per week, defined as >4 drinks for women, ≥5 drinks for men (where a single standard drink is defined by 10 g of ethanol). Any conflicting information will be validated by collateral reports (provided by electronic medical records or clinicians) and measurement of biological markers of alcohol consumption (PEth). We will also examine absence of any HDDs per week over treatment period and standard drinks per drinking day (DDD).

##### Additional clinical outcomes

We will also examine change in other clinical conditions and symptoms listed above including depression (DASS), sleep disturbance (ISI), health-related quality of life (SF-36) PTCI.

##### Safety outcomes

Safety will be analysed using categorical outcomes, defined by the type and severity of AEs assessed throughout the trial (in addition to ongoing measures of suicidality and mood as listed above). AEs will also be systematically recorded as per the COMBINE SAFTEE tool in the week following each dosing.

##### Feasibility outcomes

Engagement will be examined via the treatment satisfaction assessments (YES, CSQ) plus treatment retention and adherence. Uptake will be examined via recruitment rate (participants/month/site), screening-to-enrolment ratio and the reasons for exclusion/refusal.

### Sample size calculations

This study is sufficiently powered to estimate the effects of COPE+MDMA versus COPE+Control on significant reductions in clinician-administered PTSD symptom severity over time (CAPS-5). For the COPE+Control, based on our previous COPE trial^17^ we assume a 26% reduction in CAPS-5 score from baseline to treatment completion (Week 14) and a 42% reduction from baseline to longer follow-up. For COPE+MDMA, based on prior work of MDMA-assisted therapy,^71 72^ we assume a 55%–63% reduction from baseline. We consider these estimated separations of CAPS-5 data over repeated measures. To achieve >80% power for a two-sided test at α=0.05 conservatively assuming a within-participant correlation of 0.5 and 15% data loss, based on prior studies,^47^ 50 participants will be needed per arm (active vs control) (n=100) for the effect of MDMA versus control, time and treatment×time where the groups separate to a medium to large effect (f=0.25, Cohen’s d=0.5–0.7).

### Statistical analysis

All available participant data will be employed to follow intention-to-treat principles. Participants will be followed irrespective of whether they continue to receive treatment. We obtained a retention rate of ~90% for assessments in our most recently completed alcohol pharmacotherapy trial.^47^ Nonetheless, as with all alcohol and/or PTSD treatment studies, we anticipate intermittent missing data which will be handled through use of multiple imputation (if appropriate, following investigation of the mechanism causing data missingness, see Jakobsen *et al*^75^) and assessed via sensitivity analysis. MDMA versus control will first be compared over repeated measures of the primary outcome, CAPS-5 score, followed by the secondary outcome, HDD frequency/week, using mixed effects models for continuous and count responses respectively (models can also be extended to allow for zero inflation where required). Other outcomes will also be examined using mixed models, adjusted for multiple comparisons. Additional covariates and predictors will be included for sensitivity analyses. Similarly, as per our prior work,^43 76^ we will also examine predictors of response (eg, sex, treatment expectancy, severity of AUD and/or PTSD).

### Cost-effectiveness analysis

We will conduct an economic evaluation alongside the current RCT. This will involve a cost-effectiveness analysis that will be conducted from both the health sector and societal perspectives. As per prior work,^77^ the primary outcome will comprise Quality-Adjusted Life Years (QALYs) calculated using the SF-6D (adapted from 11 SF-36 items) at baseline and follow-up. The Health Service Use Questionnaire our team has developed will capture additional resource use such as health professional visits, hospitalisations and lost productivity. Standardised economic evaluation techniques including incremental analysis of mean differences and bootstrapping to determine CIs will be used.^78^ If the intervention is found to be cost-effective under trial conditions, then modelling techniques will be used to estimate the cost-effectiveness and budget impact of scaling up the intervention across the relevant comorbid population in Australia.

## Discussion

The MPATHY study will be the first double-blind RCT to examine the therapeutic and cost-effectiveness of evidence-based (1) integrated exposure therapy for PTSD and AUD+MDMA versus (2) integrated exposure therapy+active control in improving PTSD and alcohol outcomes. The trial will also examine the effectiveness of integrated exposure therapy+MDMA on a range of salient comorbid conditions and symptoms associated with the comorbidity such as depression, sleep disturbances and quality of life, as well as treatment satisfaction, engagement and uptake. We will also thoroughly examine AEs throughout. This report has been prepared in accordance with the SPIRIT protocol for reporting of clinical trial protocols.^35^

Results of the MPATHY study will add to the current literature given it will be the first, well-powered, double-blind, randomised comparison of integrated exposure therapy for PTSD and AUD (ie, COPE) with MDMA versus with active control. The secondary outcomes and comprehensive evaluation of AEs to be evaluated in this trial will provide clinically important information about clinical groups that are likely to benefit from this treatment. While the use of an objective laboratory marker of alcohol use is not novel it is recommended for all alcohol treatment trials,^79^ and will be the first MDMA trial to use PEth for this purpose. It is also the first cost-effectiveness analysis of MDMA for the treatment of AUD+PTSD. A critical strength of this project is also that it leverages an evidence-based behavioural intervention with strong empirical support for all participants. Notwithstanding, MDMA-assisted sessions within a manualised COPE framework (MPATHY intervention) are relatively resource-intensive, requiring specialised staff and extended sessions. Feasibility and scalability, particularly in routine clinical settings, will thus be explored alongside cost-effectiveness analyses. These outcomes will be highly relevant for generalisability to low resource settings.

It has been suggested that the effect sizes in MDMA trials have been overestimated in previous studies due to poor methodology^34^ including: (1) strong expectancy effects; (2) sample sizes are small leading to wide variation in estimates; (3) objective outcome measures are rarely used; (4) failure to maintain blinding of participants (with few studies assessing and reporting on blinding); (5) poor control with regards to which specific therapeutic elements are used throughout therapy and which different elements are impactful and (6) no inclusion of gold-standard evidence-based therapeutic comparators. Accordingly, we will conduct a double-blind RCT that includes an active control, prospective stratification for expectancy, objective alcohol outcome measures, comprehensive evaluation of AEs, independence from commercial interest and partnership with advocacy groups and, importantly, utilisation of specific evidence-based therapeutic modalities for comorbid PTSD and AUD as the ‘base’ therapy.

Improving the therapeutic benefit of integrated exposure therapy for PTSD+AUD remains a challenge particularly given the presence of comorbidities that may be contraindicated for certain medications such as MDMA. The MPATHY trial thus has the potential to facilitate the development of alternate options to optimise integrated exposure therapy but also to add to the literature regarding the suitability of MDMA-assisted therapy for this comorbid population in light of existing, safe, evidence-based gold-standard therapies.

## Supplementary material

10.1136/bmjopen-2025-114896 online supplemental file 1
