# Supplementary material for: MDMA-assisted PTSD and Alcohol Therapy Trial (MPATHY): study protocol for a double-blind, randomised, controlled outpatient trial of MDMA-assisted integrated exposure-based therapy for comorbid post-traumatic stress disorder and alcohol use disorder
Source: BMJ Open. 2026 Jul 6;16(7):e114896. doi: 10.1136/bmjopen-2025-114896 (PMC13343076; doi:10.1136/bmjopen-2025-114896)
Supplement: online supplemental file 1 [file bmjopen-16-7-s001.docx]

**Supplementary Material**

## Table 1. Schedule of Assessments

| Visit (V) Types  Screening (SCN), Baseline (BL), COPE, Preparation IPREP) Dose, Integration (INT), Research & Medical (RM), End of Treatment (EOT), Follow-up (F) | | V0 | |  | V1-3 | V4 | V5 | | V6-7 | V8 | V9-10 | V11 | V12 | | V13-15 | V16 | V17 & 18 |
| --- | --- | --- | --- | --- | --- | --- | --- | --- | --- | --- | --- | --- | --- | --- | --- | --- | --- |
|  |  | SCN |  | BL | COPE | PREP,  RM | Dose | INT | COPE | COPE, RM | COPE | PREP, RM | Dose | INT | COPE | COPE, EOT | F |
| Informed Consent | | **X** |  |  |  |  |  |  |  |  |  |  |  |  |  |  |  |
| BP, HR, ECG, height, weight | | **X** |  | ***X^4^*** |  |  |  |  |  |  |  |  |  |  |  |  |  |
| CMs, AEs^1^, major stressors^6^ | | **X** |  | ***X^4^*** | **X** | **X** | **X** | **X** | **X** | **X** | **X** | **X** | **X** | **X** | **X** | **X** | **X** |
| Medical Examination & Review | | **X** |  | ***X^4^*** |  | **X** | **X** |  |  |  |  | **X** | **X** |  |  | **X^2^** | **X^2^** |
| Pregnancy test^2^, urine drug testing, BrAC | | **X** |  | ***X^4^*** |  |  | **X** |  |  |  |  |  | **X** |  |  |  |  |
| Demographics, ethnicity, socioeconomics | |  |  | **X** |  |  |  |  |  |  |  |  |  |  |  |  |  |
| Formulation Session | |  |  | **X** |  |  |  |  |  |  |  |  |  |  |  |  |  |
| Preparation Session | |  |  |  |  | **X** |  |  |  |  |  | **X**^5^ |  |  |  |  |  |
| COPE Sessions – 12 in Total | |  |  |  | **X** |  |  |  | **X** | **X** | **X** |  |  |  | **X** | **X** |  |
| Dosing Session (6h) – BP, HR, BT | |  |  |  |  |  | **X** |  |  |  |  |  | **X** |  |  |  |  |
| Integration Session | |  |  |  |  |  |  | **X** |  |  |  |  |  | **X** |  |  |  |
| Regular Phone Check-Ins Post-Dosing | |  |  |  |  |  |  | **X** |  |  |  |  |  | **X** |  |  |  |
| Questionnaires/Measurements | CIWA-Ar, C-SSRS-L, LEC-5, MoCA, SCID-5 | **X** |  |  |  |  |  |  |  |  |  |  |  |  |  |  |  |
|  | CAPS-5 | **X** |  | ***X^4^*** |  |  |  |  |  |  |  |  |  |  |  | **X** | **X** |
|  | TLFB | **X** |  | **X*^4^*** | **X** | **X** |  |  | **X** | **X** | **X** | **X** |  |  | **X** | **X** | **X** |
|  | C-SSRS-R, PCL-5 | **X** |  | ***X^4^*** | **X** | **X** |  |  | **X** | **X** | **X** | **X** |  |  | **X** | **X** | **X** |
|  | Alcohol Consumption, PANAS^3^ – For 7 Days |  |  | **X** |  |  |  | **X** |  |  |  |  |  | **X** |  |  |  |
|  | ADS, DASS, HSUQ, ISI, PTCI, SF-36 |  |  | **X** |  |  |  |  |  | **X** |  |  |  |  |  | **X** | **X** |
|  | NEO PI-3, TMT |  |  | **X** |  |  |  |  |  |  |  |  |  |  |  | **X** |  |
|  | ACEs, SETS |  |  | **X** |  |  |  |  |  |  |  |  |  |  |  |  |  |
|  | CVLT3, ESCWT, NLT |  |  |  | **X7** |  |  |  |  |  |  |  |  |  |  | **X** |  |
|  | SRS |  |  |  | **X** |  |  | **X** | **X** | **X** | **X** |  |  | **X** | **X** | **X** |  |
|  | HAQ-II – participant and therapist |  |  |  |  | **X** |  |  |  | **X** |  | **X** |  |  |  | **X** |  |
|  | MEQ30, Mood (Likert Scale; hourly) |  |  |  |  |  | **X** |  |  |  |  |  | **X** |  |  |  |  |
|  | PMQ-SF |  |  |  |  |  |  | **X** |  |  |  |  |  | **X** |  |  |  |
|  | CSQ-8, YES |  |  |  |  |  |  |  |  |  |  |  |  |  |  | **X** |  |
| Bloods | FBC, LFT, Coagulation | **X** |  |  |  |  |  |  |  | **X** |  |  |  |  |  | **X** |  |
|  | PEth and other biomakers |  |  | **X** |  |  |  |  |  | **X** |  |  |  |  |  | **X** |  |
|  | Blood sample for biomarkers |  |  | **X** |  |  |  |  |  |  |  |  |  |  |  | **X** |  |

**Notes.**

Abbreviations: ACEs = Adverse Childhood Experiences, SCN = screening, ADS = Alcohol Dependence Scale, AE = Adverse Event, BL = Body Temperature, BrAC = Breath Alcohol Content, CAPS-5 Clinician-Administered PTSD Scale for DSM-5, CIWA-Ar = Clinical Institute Withdrawal Assessment for Alcohol, CM = Concomitant Medication, COPE = Concurrent treatment of PTSD and substance use disorders using prolonged exposure, C-SSRS-L/ R = Columbia-Suicide Severity Rating Scale-Lifetime/Recent, CSQ-8 Client Satisfaction Questionnaire – 8 CVLT-3 = California Verbal Learning Test, DASS = Depression, Anxiety & Stress, Emotional Stroop Colour Word Task = ESCWT, FBC = Full Blood Count, HAQ-II = Helping Alliance Questionnaire II, HSUQ, = Health Service Use Questionnaire, ISI = Insomnia Severity Index, LEC-5 = Life Events Checklist for DSM-5, LFT = Liver Function Tests_,_ MEQ30 = Mystical Experience Questionnaire, MoCA = Montreal Cognitive Assessment, MRI = Magnetic Resonance Imaging, NEO-PI-3 = NEO Personality Inventory 3, NLT = Number Letter Task, PANAS = Positive and Negative Affect Schedule, PCL-5 = PTSD Checklist for DSM-5, PEth = Phosphatidylethanol, PMQ-SF = Psychedelic Music Questionnaire – short form, PREP = Preparation session, PTCI = Post Traumatic Cognitions Inventory, SCID-5 = Structured Clinical Interview for DSM-5, SF-36 = Short Form 36, SRS = Session Rating Scale, TMT = Trail Making Task, TLFB = Time Line Follow Back, Your Experience of Service = YES.

1 = Not at SCN, 2 = Where indicated, 3 = Completed 3x daily, 4 = Repeated if BL and SCN is different days**,** 5= Completed in previous

COPE visit if appropriate, 6 = Only asked at COPE sessions, 7 = After COPE Session 1
