# Supplementary material for: MDMA-assisted PTSD and Alcohol Therapy Trial (MPATHY): study protocol for a double-blind, randomised, controlled outpatient trial of MDMA-assisted integrated exposure-based therapy for comorbid post-traumatic stress disorder and alcohol use disorder
Source: BMJ Open. 2026 Jul 6;16(7):e114896. doi: 10.1136/bmjopen-2025-114896 (PMC13343076; doi:10.1136/bmjopen-2025-114896)
Supplement: Supplementary file 2 [file bmjopen-2025-114896.reviewer_comments.pdf]

PEER REVIEW HISTORY

BMJ Open publishes all reviews undertaken for accepted manuscripts. Reviewers are asked to complete a checklist review form and are provided with free text boxes to elaborate on their assessment. These free text comments are reproduced below.

ARTICLE DETAILS

Title (Provisional)

The MDMA-assisted PTSD and Alcohol THerapy Trial (MPATHY): Study protocol for a double-blind, randomised, controlled outpatient trial of MDMA-assisted integrated exposure-based therapy for comorbid post-traumatic stress disorder and alcohol use disorder

Authors

Morley, Kirsten C; Arunogiri, S.; Mills, K.; Watt, J.; Teesson, M.; Baillie, A.; Lee, Y. Y.; Morse, A.; Back, S.E.; Lubman, D.I.; Haber, P. S.

VERSION 1 - REVIEW

**Reviewer**	**1**
**Name**	**Ainamani, Herbert**
**Affiliation**	**Kabale University School of Medicine, Psychiatry**
**Date**	**22-Mar-2026**
**COI**	**None**

General Overview

Thank you for proving me with the opportunity to reviwe this manuscript (RCT) that seeks to evaluates the efficacy, safety, and cost-effectiveness of MDMA-assisted integrated exposure-based therapy for individuals with comorbid PTSD and AUD). The study addresses a clinically significant and complex comorbidity treatment approaches that adds to clinical scientif rigor. However, there are some issues that need to be addressed before its publication.

Abstract

• Can the authors briefly clarify the novelty of the trial (e.g., "first double-blind RCT combining MDMA with COPE") to better highlight its contribution.

• The abstract could also benefit from a brief mention of sample size to improve completeness. The primary aim of the MPATHY ……………..(Since this is the first mention of this acronym, can the authors explain in parentheses).

Introduction

The introduction provides a strong and coherent rationale for the study. The burden of PTSD–AUD comorbidity and limitations of existing treatments are clearly articulated. How the the authors could,

• Acknowledge more explicitly that mechanistic pathways (e.g., fear extinction, amygdala modulation) are not directly measured in this trial, and position this as an area for future research.

Methods

Study Design

• There is a high likelihood of functional unblinding due to the psychoactive effects of MDMA. Can the authors provide more detail on how blinding integrity will be assessed and incorporated into the statistical analysis.

Participants (Inclusion and Exclusion Criteria)

• The requirement that participants must have received prior treatment for PTSD or AUD requires clarification. The authors should specify what constitutes “prior treatment” and justify this criterion. I am worried this couls introduce selection bias toward treatment-resistant populations, Thus limiting generalizability.

Intervention

Althpough the integration of MDMA-assisted sessions within a manualised COPE framework is well described, the intervention is resource-intensive, requiring specialized staff and extended sessions. Can the authors briefly discuss feasibility and scalability, particularly in routine clinical settings.

Outcomes and Measures

• The authors are commended for coming up with cormbidities. They would, however, need to clarify how multiple outcomes will be prioritised or adjusted for multiplicity in the analysis.

Discussion

The discussion appropriately highlights the significance and innovation of the study. The authors could expand the discussion to real-world applicability and implementation challenges, especially in low-resource settings and further elaborate more on generalizability, given the strict eligibility criteria and controlled trial conditions.

Over all conclusion

This protocol outlines a highly innovative and methodologically rigorous trial addressing a critical gap in the treatment of comorbid PTSD and AUD. The integration of MDMA-assisted therapy with an evidence-based psychotherapeutic framework is a significant strength.

**Reviewer**	**2**
**Name**	**Bischof, Gallus**
**Affiliation**	**University of Lubeck, Psychiatry and Psychotherapy**
**Date**	**30-Mar-2026**
**COI**	**None**

This is an interesting approach to evaluate if MDMA-assisted psychotherapy might improve outcomes in individuals with Alcohol Use Disorders and PTSD. Overall, the rationale provided seems conclusive and the study design is well organized. However, there are some issues that in my understanding need to be clarified:

Background: Literature on the efficacy of MDMA for the treatment of PTSD should be cited (e.g. Sharour et al., 2024)

Inclusion/Exclusion criteria: I wonder why participants should be treatment naive while previous MDMA or selective serotonine reuptake inhibitor use is recorded but not treated as an exclusion criteria (especially since it is likely that experienced participants will be well aware of their treatment condition).Although regular users of MDMA are excluded, i assume that a lifetime-experience should be sufficient to unravel blinding for participants. In addition, i assume that adding this as an exclusion criterion will not cause any recruitment problems.

Intervention procedure: „Abstinence from Alcohol will be encouraged but not required“: i understand that you will assess alcohol use using TLFB. I wonder if there are any safety plans in case of interactions with medication. I also wondered why testing for alcohol using a breathalyzer and routine drug screening is not part of your procedure.

**Reviewer**	**3**
**Name**	**Feeny, N**
**Affiliation**	**Case Western Reserve University, Psychological Sciences**
**Date**	**30-Mar-2026**
**COI**	**None**

BMJ Open

Title: The MDMA-assisted PTSD and Alcohol THerapy Trial (MPATHY): Study protocol for a double-blind, randomised, controlled trial of MDMA-assisted integrated exposure based therapy for comorbid post-traumatic stress disorder and alcohol use disorder

Authors: Morely et al.

This paper described the protocol for a double-masked RCT evaluating MDMA-assisted exposure therapy (COPE) versus COPE paired with an active control (niacin) for treating comorbid PTSD and AUD. Overall, the protocol is well-written, as it clearly describes each step of the methodology in sufficient detail. Further, it is evident that this methodology has been carefully considered and much attention to detail has been paid with regards to precautions for safeguarding participant well-being. Importantly, the design of this trial also specifically addresses important limitations of previous trials examining the use of psychedelics to augment treatment for PTSD and related conditions. Suggestions for minor revisions of this article are described below.

1. The authors clearly and succinctly summarized the literature on PTSD, AUD, their common co-occurrence, and their most effective treatment to date. However, the rationale presented for using MDMA to assist psychotherapy does not appear to be sufficiently supported in the introduction. The previous studies cited in support of the claim that increases in monoamine neurotransmitters and hormones caused by MDMA lead to feelings of closeness and trust, which the authors then assert may strengthen therapeutic alliance, are two studies conducted with rats that were published over 20 years ago. There has been a good deal of work done in this area with humans (both outside of and within therapeutic contexts) over the last decade, and it would be important for the present study to refer to this work. It would likely also be helpful to refer to the body of process-based psychotherapy literature on the topic of therapeutic alliance. The authors' argument that MDMA may enhance memory processing and fear extinction could also benefit from additional support. Given that MDMA is not administered every session, it seems particularly important to explain this process a bit more thoroughly and whether/how the benefits of MDMA may generalize to patients' abilities to approach and either habituate to or tolerate trauma-related distress beyond the context of being under the influence of the drug.

2. On p.4, the authors assert that people with PTSD-AUD comorbidity experience a "more severe and complex form of PTSD characterized by extensive trauma histories." I might consider rephrasing because it seems to imply that people with this comorbidity always have extensive trauma histories and higher PTSD severity, which is not necessarily the case. Also, the citations here appear to refer to work in populations with PTSD and heroin dependence, rather than alcohol, so it would be helpful to provide specific references related to populations with AUD given the focus of the present study.

3. It would be helpful to provide more detail about the "integration sessions" in the methods section (e.g., How are they structured - do they follow any specific format? Are the techniques used in this session drawn from any evidence-based approaches?). It could also be beneficial to introduce the concept of integration earlier (e.g., in the introduction), citing previous examples of therapies that have included these sessions and providing explanation for why these sessions are needed, particularly for audiences who are less familiar with psychedelic-assisted approaches.

4. Because one of the inclusion criteria is having received prior treatment for PTSD or AUD, it would be helpful to include a bit of rationale for why this is the case. I am guessing that the reason may be to target patients for whom traditional treatments have not been effective, but it would be beneficial to say this explicitly.

5. Regarding the exclusion criteria of other substance use disorder besides tobacco, does this mean just current diagnosis or having any history of SUD? I see that recent/current ecstasy use is also an exclusion criterion, though I wonder about folks with SUD histories (particularly those related to ecstasy and amphetamines) so clarification around this would be helpful.

6. Do participants undergo screening on the day of dosing sessions? Given that abstinence from alcohol is encouraged but not required, and that this is a population with AUD, what is the protocol for situations in which a patient presents to a dosing session intoxicated?

7. The policies of requiring two people to always be present in the room during dosing sessions and audio/video recording these sessions is commendable from an ethical perspective!

8. On p.22, an "Aim 1" is mentioned but I was unable to find where Aim 1 was initially introduced, so clarification about aims is warranted.

9. A major strength of this protocol, explained very well in the Discussion, is the care with which the protocol was designed to address important limitations of prior work in the area of psychedelic-assisted therapy (e.g., double-blind design; the use of an evidence-based behavioral intervention with strong empirical support, in both treatment conditions; sufficient sample size; controlling for expectancy effects; and inclusion of objective outcome measurements). These methodology considerations will offer significant weight to this study's findings.

10. The Consumer Engagement section on p.28 mentions a "lived experience investigator" and a "lived experience research assistant." It was unclear what type of lived experience these individuals have.

11. I recommend an additional review of the article for minor spelling/grammar errors.

VERSION 1 - AUTHOR RESPONSE

Reviewer 1

Abstract

RC: Can the authors briefly clarify the novelty of the trial (e.g., "first double-blind RCT combining MDMA with COPE") to better highlight its contribution.

AR: This has been done (Abstract: first sentence in Methods; final sentence of Abstract; first sentence in the Discussion)

RC: The abstract could also benefit from a brief mention of sample size to improve completeness.

AR: This has been done.

RC: The primary aim of the MPATHY ………………(Since this is the first mention of this acronym, can the authors explain in parentheses).
AR: This is noted with thanks and it has been done.
RC: Acknowledge more explicitly that mechanistic pathways (e.g., fear extinction, amygdala modulation) are not directly measured in this trial, and position this as an area for future research.
AR: This has been done (Page 7).
RC: There is a high likelihood of functional unblinding due to the psychoactive effects of MDMA. Can the authors provide more detail on how blinding integrity will be assessed and incorporated.
AR: At the end of the first paragraph in Methods in the Medications section we detail assessment of double blinding, how it will be assessed and incorporated (noting that CONSORT guidelines preclude this being incorporated into primary analyses whereby it must remain as a sensitivity analysis).
RC: The requirement that participants must have received prior treatment for PTSD or AUD requires clarification. The authors should specify what constitutes "prior treatment" and justify this criterion. I am worried this couls introduce selection bias toward treatment-resistant populations, Thus limiting generalizability.
AR: We have now provided more detail regarding examples of prior treatment for PTSD and AUD. Nonetheless, this should not be of concern given that the indication for use of MDMA for PTSD in Australia is *only* for treatment resistant populations. It is not considered as a front-line treatment.
RC: Althpough the integration of MDMA-assisted sessions within a manualised COPE framework is well described, the intervention is resource-intensive, requiring specialized staff and extended sessions. Can the authors briefly discuss feasibility and scalability, particularly in routine clinical settings.
AR: This is a great point and we agree. This is exactly why we have included a cost-effectiveness analysis which will be part of the outcomes of this study. We have now added a brief discussion on feasibility and scalability in the Discussion section (Page 26) plus specifically outlining these outcomes in the Methods (Page 23).
RC: The authors are commended for coming up with cormbidities. They would, however, need to clarify how multiple outcomes will be prioritised or adjusted for multiplicity in the analysis.
AR: We have listed several comorbid conditions to be examined as secondary analyses but identified a priori. We have also noted that we will adjust for multiple comparisons.
RC: The authors could expand the discussion to real-world applicability and implementation challenges, especially in low-resource settings and further elaborate more on generalizability, given the strict eligibility criteria and controlled trial conditions.
AR: We thank the reviewer for this input and we have now added this in the discussion (Page 26).

RC: Literature on the efficacy of MDMA for the treatment of PTSD should be cited (e.g. Sharour et al., 2024)
AC: We now also include the suggested citation.
RC: I wonder why participants should be treatment naive while previous MDMA or selective serotonine reuptake inhibitor use is recorded but not treated as an exclusion criteria (especially since it is likely that experienced participants will be well aware of their treatment condition).Although regular users of MDMA are excluded, i assume

that a lifetime-experience should be sufficient to unravel blinding for participants. In addition, i assume that adding this as an exclusion criterion will not cause any recruitment problems.
AC: As stated in the protocol, participants should not be treatment naive, they are required to have received previous treatment for each condition. SSRI is an exclusion criteria. Those with prior SSRI use is recorded and stratified/balanced because they need to be weened off it to be involved. Previous MDMA use is also stratified/balanced so to keep this balanced across groups in case of blinding.
RC: Abstinence from Alcohol will be encouraged but not required“: i understand that you will assess alcohol use using TLFB. I wonder if there are any safety plans in case of interactions with medication. I also wondered why testing for alcohol using a breathalyzer and routine drug screening is not part of your procedure.
AC: As stated in the protocol, we assess for alcohol using TLFB but also PEth. PEth is a gold standard objective biomarker of alcohol use. This is part of the routine procedure. As for interactions, patients are breathlysed when they arrive for dosing and are not able to be dosed if intoxicated (this has been now explicitly stated) and the dosing session will be postponed. After dosing they remain on the premises for the day which precludes interactions with alcohol given they will not receive alcohol during the dosing day.
RC:The rationale presented for using MDMA to assist psychotherapy does not appear to be sufficiently supported in the introduction. The previous studies cited in support of the claim that increases in monoamine neurotransmitters and hormones caused by MDMA lead to feelings of closeness and trust, which the authors then assert may strengthen therapeutic alliance, are two studies conducted with rats that were published over 20 years ago. There has been a good deal of work done in this area with humans (both outside of and within therapeutic contexts) over the last decade, and it would be important for the present study to refer to this work. It would likely also be helpful to refer to the body of process-based psychotherapy literature on the topic of therapeutic alliance. The authors' argument that MDMA may enhance memory processing and fear extinction could also benefit from additional support. Given that MDMA is not administered every session, it seems particularly important to explain this process a bit more thoroughly and whether/how the benefits of MDMA may generalize to patients' abilities to approach and either habituate to or tolerate trauma-related distress beyond the context of being under the influence of the drug.
AC: We have now added two human studies as evidence for the statements regarding MDMA leading to feelings of closeness and trust and also MDMA and PTSD. We have also now added more references regarding the statement that MDMA may enhance memory processing and fear extinction. While not dosed every session, two dosing sessions followed by psychotherapy close after would be sufficient to benefit from the enhancement of these processes.
RC: On p.4, the authors assert that people with PTSD-AUD comorbidity experience a "more severe and complex form of PTSD characterized by extensive trauma histories." I might consider rephrasing because it seems to imply that people with this comorbidity always have extensive trauma histories and higher PTSD severity, which is not necessarily the case. Also, the citations here appear to refer to work in populations with PTSD and heroin dependence, rather than alcohol, so it would be helpful to provide specific references related to populations with AUD given the focus of the present study.
AC: This is noted with thanks. We have now rephrased the statement to read "can" and "often" given that, while the population level data suggest these are the associations, there are indeed individuals with this comorbidity where this is not the case.
RC: It would be helpful to provide more detail about the "integration sessions" in the

methods section (e.g., How are they structured - do they follow any specific format? Are the techniques used in this session drawn from any evidence-based approaches?). It could also be beneficial to introduce the concept of integration earlier (e.g., in the introduction), citing previous examples of therapies that have included these sessions and providing explanation for why these sessions are needed, particularly for audiences who are less familiar with psychedelic-assisted approaches.
RC: This is noted with thanks. We have now introduced the concept of integration earlier (Page 6 in the introduction) and included a little more detail regarding the integration sessions in the methods.
RC: Because one of the inclusion criteria is having received prior treatment for PTSD or AUD, it would be helpful to include a bit of rationale for why this is the case. I am guessing that the reason may be to target patients for whom traditional treatments have not been effective, but it would be beneficial to say this explicitly.
AC: Yes agreed. This is noted with thanks, Psychedelic assisted therapy is not targeted as a front line treatment. In Australia it is limited to those that are treatment-resistant. We have now provided a brief rationale.
RC. Regarding the exclusion criteria of other substance use disorder besides tobacco, does this mean just current diagnosis or having any history of SUD? I see that recent/current ecstasy use is also an exclusion criterion, though I wonder about folks with SUD histories (particularly those related to ecstasy and amphetamines) so clarification around this would be helpful.
AC: We thank the reviewer for pointing this out. We have now clarified and provided more detail regarding the SUD diagnosis (current not history). We have cited DSM-V so that should be clear to the reader that any SUD within the last 12 months will be excluded. Significant recent MDMA use is excluded not for reasons of research engagement and integrity (e.g. dependence) and safety (e.g. interactions) but due to the issues with blinding and potentially blunted action. To that end, this is why previous use of MDMA is stratified/balanced.
RC: Do participants undergo screening on the day of dosing sessions? Given that abstinence from alcohol is encouraged but not required, and that this is a population with AUD, what is the protocol for situations in which a patient presents to a dosing session intoxicated?
AC: We do BrAC readings before dosing whereby 0.00 is mandatory and higher readings lead to a postponed session. This has now been made clear in the protocol. We thank the reviewer for pointing this out.
RC: On p.22, an "Aim 1" is mentioned but I was unable to find where Aim 1 was initially introduced, so clarification about aims is warranted.
AC: This is an error from a grant application and has been deleted.
RC: The Consumer Engagement section on p.28 mentions a "lived experience investigator" and a "lived experience research assistant." It was unclear what type of lived experience these individuals have.
AC: This has now been clarified.
RC. I recommend an additional review of the article for minor spelling/grammar errors.
AC: We have run a final spell check.

VERSION 2 - REVIEW

Reviewer 1

Name Ainamani, Herbert

Affiliation Kabale University School of Medicine, Psychiatry

Date 19-May-2026

COI

The authors have adequately responded to my comments raised during the review

---

VERSION 2 - AUTHOR RESPONSE

Reviewer: 1

RC: The authors have adequately responded to my comments raised during the review

AR: Thankyou
